# The Performance of Direct Disk Diffusion for Community Acquired Bacteremia due to Gram-Negative Bacilli and Its Impact on Physician Treatment Decisions

**DOI:** 10.1155/2016/5493675

**Published:** 2016-04-06

**Authors:** Peter Daley, Adam Comerford, Jurgienne Umali, Carla Penney

**Affiliations:** ^1^Faculty of Medicine, Memorial University of Newfoundland and Labrador, Room 1J421, 300 Prince Philip Drive, St. John's, NL, Canada A1B 3V6; ^2^Faculty of Medicine, Memorial University of Newfoundland and Labrador, St. John's, NL, Canada A1B 3V6

## Abstract

*Background.* Direct disk diffusion susceptibility testing provides faster results than standard microtitre susceptibility. The direct result may impact patient outcome in sepsis if it is accurate and if physicians use the information to promptly and appropriately change antibiotic treatment.* Objective.* To compare the performance of direct disk diffusion with standard susceptibility and to consider physician decisions in response to these early results, for community acquired bacteremia with Gram-negative Bacilli.* Methods.* Retrospective observational study of all positive blood cultures with Gram-negative Bacilli, collected over one year. Physician antibiotic treatment decisions were assessed by an infectious diseases physician based on information available to the physician at the time of the decision.* Results.* 89 bottles growing Gram-negative Bacilli were included in the analysis. Direct disk diffusion agreement with standard susceptibility varied widely. In 47 cases (52.8%), the physician should have changed to a narrower spectrum but did not, in 18 cases (20.2%), the physician correctly narrowed from appropriate broad coverage, and in 8 cases (9.0%), the empiric therapy was correct.* Discussion.* Because inoculum is not standardized, direct susceptibility results do not agree with standard susceptibility results for all drugs. Physicians do not act on direct susceptibility results.* Conclusion.* Direct susceptibility should be discontinued in clinical microbiology laboratories.

## 1. Introduction

Sepsis is associated with a mortality rate of 35.3% [1], with more than eight million lives lost globally per year [2]. Early appropriate antibiotic therapy for Gram-negative bacteremia is associated with reductions in mortality [3] and length of hospital stay [4].

Sepsis treatment should begin with broad spectrum empiric antibiotic therapy and then promptly deescalate as guided by antimicrobial susceptibility results [5]. Deescalation is associated with a reduction in 28-day mortality (OR = 0.37, *p* = 0.04) in a retrospective study [6].

Direct disk diffusion susceptibility testing of positive blood cultures provides earlier susceptibility results than standard susceptibility performed according to CLSI guidelines (24 hours from detection as compared to 48 hours from detection). Because inoculum is not standardized, direct susceptibility results may not agree with the standard method, in which inoculum is standardized. One previous report demonstrated only 82.4% agreement between direct susceptibility and standard susceptibility among Gram-negative Bacilli, with poor agreement observed with cefuroxime (71.1%, with a very major error rate of 6.7%) and amoxicillin/clavulanate (60.4%, with a very major error rate of 6.8%) [7].

Early susceptibility results may impact patient outcome, but the results must be acted on appropriately and quickly by the physician in order for this benefit to be realized. In a randomized study of 251 patients with bacteremia in which rapid molecular susceptibility was provided within six hours from detection, the rapid results agreed with the standard susceptibility in 94% of patients. The rapid results were available fifteen hours earlier than standard results, but physicians only changed therapy based on rapid results in 16/129 (12.4%) patients. No benefit of rapid susceptibility results on clinical outcome was able to be demonstrated [8].

We designed a retrospective study to consider the performance and impact on physician behavior of direct disk diffusion testing for community acquired Gram-negative bacteremia in a university medical microbiology laboratory.

## 2. Methods

With local ethics approval, all blood cultures received between January 1 and December 31, 2014, were accessed. Identifying information was removed. To be included, the positive blood cultures had to be collected in an emergency department and growing Gram-negative bacteria. There were no exclusion criteria based on age or demographics.

Blood culture was performed using the BACTEC FX system (Becton Dickinson, USA). Direct susceptibility for 19 antibiotics was performed on positive blood culture bottles (aerobic or anaerobic) after Gram stain results by inoculating 2–4 drops of broth from the blood culture bottle onto two large Mueller-Hinton plates (Oxoid, Canada). The broth was swabbed onto the media in three planes and antibiotic disks were placed directly onto plates using two 12 disk inoculators. Direct susceptibility was inoculated throughout the day as bottles became positive and was read after 24 hours of aerobic incubation. Interpretive breakpoints were selected based on the direct oxidase result (Enterobacteriaceae breakpoints for oxidase negatives,* Pseudomonas* breakpoints for oxidase positives). Bacterial identification and standard susceptibility were performed using Siemens MicroScan GNUC51 panel (Siemens, USA) which served as the reference susceptibility method. Susceptibility breakpoints and extended spectrum beta-lactamase (ESBL) confirmation using Kirby Bauer followed CLSI M100-S25 (2015).

Our testing policy is to perform direct disk diffusion on Gram-negative Bacilli detected on admission to hospital only and only on the first positive bottle. In some cases, direct susceptibility was not performed. Direct susceptibility is reported with an interpretive comment explaining that direct susceptibility may not correlate with standard susceptibility. Our laboratory does not perform testing after 1800 hours. Susceptibility results are provided to physicians using online reporting.

Correlation was reported using percent agreement. A very major error was defined as an error reporting a resistant isolate as susceptible; a major error was defined as an error reporting a susceptible isolate as resistant; a minor error was defined as an error reporting a resistant or susceptible isolate as intermediate or vice versa [9]. Intermediate results from direct disk diffusion were not reported to the physician.

Treatment decisions were retrospectively assessed by an infectious disease physician using the online patient care record, for appropriateness based on the match between the susceptibility result and the antibiotics chosen after the direct susceptibility result was provided, but before the standard susceptibility result was provided. Treatment decision categories were created based on subjective interpretation of the suitability of the choice.

Treatment decisions were assessed assuming that direct susceptibility results were accurate and that physicians received the result and made the appropriate antibiotic selection based on the result. The infectious diseases physician was not involved in the care of the patients during the first day of admission but may have been consulted after the standard susceptibility results were available.

## 3. Results

**Figure 1:**
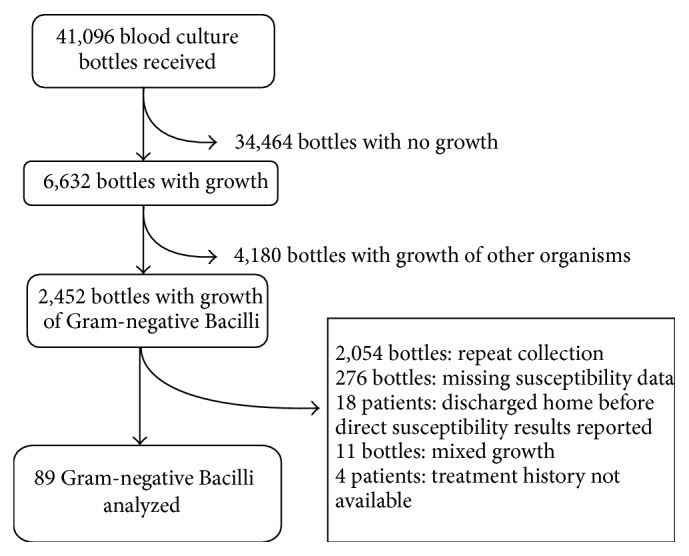
Specimen flow.

41,096 blood culture bottles were received, of which 6,632 grew bacteria (16.1% positivity). 2,452 of the positive bottles grew Gram-negative Bacilli. We excluded 2,054 repeat positives from the same collection time, 276 bottles which did not undergo direct susceptibility testing, 18 patients who were discharged home before results were released, 11 bottles with mixed growth, and 4 patients with missing treatment data, leaving a total of 89 eligible blood cultures in our analysis (Figure 1).

Gram-negative Bacilli included were predominantly* E. coli* (55 strains, 62%) and* Klebsiella pneumoniae* (13 strains, 15%). Eight strains expressed extended spectrum beta-lactamases (9%). No anaerobes were identified in the study.* E. coli* strains were predominantly susceptible to cephalosporins, beta-lactam/beta-lactamase inhibitor combinations, aminoglycosides, and fluoroquinolones, but other strains were more resistant (Table 1).

Nineteen antibiotics were tested using direct susceptibility, but in some cases direct testing was not performed. Eight antibiotics were not analyzed because they were missing more than 15 percent of comparison data, leaving 11 antibiotic comparisons.

49 patients (55.1%) were male and 40 (44.9%) were female. The mean patient age was 66 years with a standard deviation of 15.8 years, ranging from 3 to 89 years.

The correlation between direct susceptibility and standard susceptibility varied widely (Table 2). Gentamicin had the highest rate of agreement (96.6% with 0% very major or major errors), followed by Ciprofloxacin (94.4% with 0% very major or major errors) and Cefotaxime (91.0% with 1.1% very major errors). The lowest rates of agreement were seen with Cefazolin (40.0% agreement with 14.6% very major errors and 9.0% major errors) and Amoxicillin/clavulanic acid (52.8% agreement with 20.2% very major errors and 3.4% major errors).

The correlation between direct susceptibility and standard susceptibility was poor for all species (Table 3).* E. coli* (non-ESBL) demonstrated 81.6% agreement,* E. coli* (ESBL) demonstrated 81.8% agreement, and* Klebsiella pneumoniae* demonstrated 73.4% agreement.

In all 89 cases, physician treatment decisions were assessed (Table 4). In 31/89 cases (26.0%), treatment decisions were considered appropriate. 18 (20.2%) physicians correctly narrowed antibiotic therapy from broad coverage, while five (5.6%) who administered incorrect antibiotics changed to appropriate therapy, and eight (9.0%) were already on appropriate empiric therapy, which was not changed. However, 47 (52.8%) failed to change therapy from broad coverage to a narrower spectrum. Furthermore, two (2.2%) physicians continued inappropriate therapy, six (6.7%) inappropriately broadened from appropriate empiric therapy, and one (1.1%) changed from appropriate therapy to inappropriate therapy. Two (2.2%) discontinued antibiotic therapy after receipt of direct susceptibility.

## 4. Discussion

Correlation between direct and standard susceptibility is variable, meaning that in many cases the direct result is incorrect, and may actually mislead antibiotic treatment decisions. This conclusion is expected, based on the lack of inoculum standardization with the direct method. The interpretive comment in the report warns the physician that standard susceptibility may differ from direct susceptibility, and, for this reason, we observed 47 cases (52.8%) in which physicians did not change treatment based on the direct susceptibility result. This physician response is appropriate and demonstrates that the physician is unsure that the direct result is correct.

There was only a single previous report found examining the performance of direct susceptibility [7], and there are no published guidelines describing a standardized method to perform direct susceptibility. Our analysis provides evidence of inconsistent performance with direct susceptibility. We would suggest that direct susceptibility testing be discontinued in clinical microbiology laboratories. A rapid result cannot benefit the patient if the rapid result is incorrect. Furthermore, reporting a result which contains a warning comment may reduce the trust that the physician holds in the quality of the laboratory result.

Strengths of our study include an assessment of the clinical impact of lab results, as measured by physician behavior. The microbiology literature often does not often report on the clinical impact of diagnostic tests but is limited to reports of diagnostic performance [7, 10]. Treatment choice is an indirect measure of diagnostic impact. There are unmeasured factors which influence treatment decisions, such as severity of illness, source of bacteremia, patient allergies, or secondary infections, so treatment alone is not a perfect marker of diagnostic impact. We encourage the development and validation of markers of diagnostic impact in microbiology and the reporting of diagnostic impact outcomes at both the patient level (mortality, length of stay) and societal level (cost benefit, disease control) to the clinical microbiology literature.

Limitations of our study include retrospective data collection and small sample size. We do not have enough bacteria included to make conclusions based on individual species. Significant missing data based on inconsistent application of testing could have influenced our analysis but would be unlikely to influence the conclusion that the performance of direct susceptibility was variable. The strains included in the study were a very small proportion of positive blood cultures during the period of study, because direct susceptibility was performed in a very selective fashion. If direct susceptibility was performed on a larger proportion of positive blood cultures, this would also be unlikely to influence the conclusion.

## 5. Conclusions

Direct susceptibility provides variable performance and should be discontinued. Alternative molecular methods for rapid susceptibility may provide better performance. Diagnostic methods in microbiology should report patient-relevant clinical outcomes.

## Figures and Tables

**Table 1:** Susceptibility profile of included Gram-negative Bacilli.

Species (*n* = 89)	% resistant (standard susceptibility)										
	Ampicillin	Gentamicin	Ciprofloxacin	Cefazolin	Cefotaxime	Amoxicillin/clavulanic acid	Piperacillin-tazobactam	Ceftazidime	Trimethoprim-sulfamethoxazole	Imipenem	Tobramycin
*E. coli* (48)	50	2.1	12.4	8.3	2.1	4.2	0	2.1	20.8	0	2.1
*E. coli* ESBL (7)	100	28.6	100	100	100	100	100	100	57.1	0	28.6
*Klebsiella pneumoniae* (13)	100	0	7.7	100	0	92.3	0	0	15.4	0	7.7
*Proteus mirabilis* (5)	100	0	0	100	0	100	0	0	20	20	0
*Enterobacter *sp. (5)	100	20	0	100	40	100	20	40	0	0	20
*Klebsiella oxytoca* (3)	66.7	0	0	33.3	0	33.3	0	0	0	0	0
*Klebsiella oxytoca* ESBL (1)	100	0	0	100	100	100	100	0	0	0	0
*Serratia marcescens* (3)	100	0	0	100	33.3	100	0	33.3	0	0	0
*Pseudomonas aeruginosa* (3)	100	0	0	100	0	100	0	0	66.7	0	0
*Burkholderia cepacia* (1)	100	100	0	100	100	100	0	100	0	100	100

**Table 2:** Agreement between direct and standard susceptibility.

Antibiotic	Agreement (*N*/%)	Very major error rate (*N*/%) (falsely reporting resistant isolates as susceptible)	Major error rate (*N*/%) (falsely reporting susceptible isolates as resistant)	Minor error rate (*N*/%) (falsely reporting intermediate isolates as susceptible or resistant or vice versa)	Missing data (*N*/%)
Ampicillin	69 (77.5)	5 (5.6)	3 (3.4)	10 (11.2)	2 (2.2)
Gentamicin	86 (96.6)	0 (0)	0 (0)	1 (1.1)	2 (2.2)
Ciprofloxacin	84 (94.4)	0 (0)	0 (0)	3 (3.4)	2 (2.2)
Cefazolin	36 (40.4)	13 (14.6)	8 (9.0)	24 (30.0)	8 (9.0)
Cefotaxime	81 (91.0)	1 (1.1)	0 (0)	7 (7.9)	0 (0.0)
Amoxicillin/clavulanic acid	47 (52.8)	18 (20.2)	3 (3.4)	12 (13.5)	9 (10.1)
Piperacillin/tazobactam	69 (77.5)	4 (4.4)	1 (1.1)	10 (11.2)	5 (5.6)
Ceftazidime	77 (86.5)	2 (2.2)	0 (0)	2 (2.2)	8 (9.0)
Trimethoprim/sulfamethoxazole	71 (79.8)	0 (0)	4 (4.4)	5 (5.6)	9 (10.1)
Imipenem	76 (85.4)	1 (1.1)	0 (0)	1 (1.1)	11 (12.4)
Tobramycin	75 (84.3)	0 (0)	0 (0)	2 (2.2)	12 (13.5)

**Table 3:** Agreement between direct and standard susceptibility by species.

Species (*n*)	Mean agreement rate (%)	Mean very major error rate (%)	Mean major error rate (%)
*E. coli* (48)	81.6	0	3.0
*E. coli* ESBL (7)	81.8	7.8	0
*Klebsiella pneumoniae* (13)	73.4	11.9	0
*Proteus mirabilis* (5)	58.2	27.3	0
*Enterobacter* sp. (5)	81.8	1.8	0
*Klebsiella oxytoca* (3)	57.6	6.0	3.0
*Klebsiella oxytoca* ESBL (1)	100	0	0
*Serratia marcescens* (3)	81.8	3.0	0
*Pseudomonas aeruginosa* (3)	90.9	0	3.0
*Burkholderia cepacia* (1)	90.0	10	0

**Table 4:** Physician response to direct susceptibility results.

Response	Appropriate response	Frequency	Percent
No change but empiric therapy correct	Yes	8	9.0
Changed from inappropriate therapy to appropriate therapy	Yes	5	5.6
No change, continued inappropriate therapy	No	2	2.2
Changed from appropriate therapy to inappropriate therapy	No	1	1.1
Should have changed to a narrower spectrum but did not	No	47	52.8
Correctly narrowed from appropriate broad coverage	Yes	18	20.2
Inappropriately broadened from appropriate empiric therapy	No	6	6.7
Antibiotics discontinued	No	2	2.2
Total		89	100.0
